# The Establishment of National Diagnostic Reference Levels for Pediatric Chest X‐Ray Examinations in Ethiopia

**DOI:** 10.1155/rrp/4441505

**Published:** 2026-10-05

**Authors:** Leul Adane Chemeda, Seife Teferi Dellie, Getu Ferenji, Daniel Zewdneh Solomon, Marema Jebessa Kumsa, Solomon Getachew

**Affiliations:** ^1^ Department of Radiology College of Health Sciences Addis Ababa University Addis Ababa Ethiopia aau.edu.et; ^2^ Department of Internal Medicine St. Paul’s Hospital Millennium Medical College Addis Ababa Ethiopia sphmmc.edu.et; ^3^ Department of Medical Radiologic Technology College of Health Sciences Addis Ababa University Addis Ababa Ethiopia aau.edu.et; ^4^ Department of Technology and Innovation Management Adama Science and Technology University Adama Ethiopia astu.edu.et

**Keywords:** chest X-ray, dose optimization, entrance surface air kerma, national diagnostic reference levels, pediatric

## Abstract

**Background:**

It is well established that diagnostic X‐ray practices must be optimized to keep patient radiation dose as low as possible with providing the diagnostic information required. The International Commission on Radiological Protection recommends diagnostic reference levels as a practical means to optimize radiation doses in diagnostic radiology and nuclear medicine. The aim of this study was to propose national diagnostic reference levels for routine pediatric chest X‐ray examinations in Ethiopia.

**Methods:**

Anthropometric and radiographic parametric data were collected from 975 patients. A standard patient was defined based on the mean values of weight, height, and BMI for the age groups of 5 to 10 and 10 to 15 years old. The exposure parameter, entrance surface air kerma, was calculated. NDRLs for entrance surface air kerma were defined as the 75^th^ percentile of dose values.

**Results:**

The suggested national diagnostic reference levels of chest X‐ray examinations were 0.07 mGy and 0.53 mGy for age groups 5‐< 10 and 10‐< 15, respectively.

**Conclusion:**

The 75th percentile of dose values determined in this study was higher than those published in the literature. The findings reveal significant variability in ESD across hospitals, primarily due to the use of inappropriate radiological parameters. A notable inconsistency in the selection of kV and mAs parameters was also observed, underscoring the need for standardized radiological protocols.

## 1. Introduction

Diagnostic reference levels (DRLs) were established by the International Commission on Radiological Protection (ICRP) as a practical means to optimize radiation doses in diagnostic radiology and nuclear medicine [1]. Various initiatives undertaken by radiation protection organizations influenced the development of DRLs [2, 3]. DRLs represent dose levels established for typical examinations, targeting groups of standard‐sized patients and broadly defined types of equipment [4]. They serve as practical reference points and provide guidance on the appropriate dosage levels for standard radiographic procedures. Over time, DRLs have become a widely recognized and effective tool for radiation dose optimization [2].

Conventional X‐rays remain one of the most widely used imaging techniques, mainly due to their cost‐effectiveness and easy availability. Previous studies have shown that chest X‐rays (CXRs) are the most frequently requested medical procedure, accounting for nearly 25 percent of all CXR procedures [5]. Common indications for requesting CXRs in the pediatric population include cardiac disease, respiratory tract infections, gastrointestinal congenital anomalies like esophageal atresia or diaphragmatic hernia, pulmonary tuberculosis (PTB), trauma, and suspected foreign body (FB) aspiration/ingestion. It may also be requested as part of a skeletal survey or to confirm the position of peripherally inserted central catheters, the tip of the endotracheal tube, or a gastric tube [6].

The application of the DRL concept to pediatric age groups is often not extended due to several problems. One of the main barriers is the difficulty of conducting dose surveys and determining appropriate benchmarks in children. Limited data on pediatric [7, 8] radiation doses exist, as medical X‐ray procedures for children constitute a smaller proportion (< 10%) of all diagnostic X‐ray examinations [9, 10]. Despite these difficulties, it is essential to ensure dose optimization in pediatric radiographic imaging, as pediatric patients are at greatest lifetime risk of exposure to ionizing radiation. This requires strategies and measures that prioritize the achievement of the largest overall benefits. This balance is achieved by applying basic principles of radiation safety in medical practice [10].

According to a report by the UN Scientific Committee on the Effects of Atomic Radiation (UNSCEAR), children are, in about 25 percent of these cases, particularly vulnerable to radiation, including leukemia, skin, breast, and brain cancers [11]. During diagnostic imaging, children may receive disproportionately high radiation doses compared to adults unless the parameters of exposure are appropriately adjusted for this [12]. For the same amount of radiation, children are more likely to develop radiation‐induced cancers. These tumors can appear years after exposure or even decades later. Estimates of the lifetime cancer risk in children exposed to radiation are uncertain but may be 2–3 times higher than in the population as a whole [13].

However, this system can sometimes lead to unintentional underexposure or overexposure of pediatric patients [14]. This highlights the urgent need for a comprehensive radiation dose assessment in pediatric radiography in order to better understand the current situation [15–17]. To address these issues, we have conducted a national study in Ethiopia to evaluate radiation doses in CXRs of children and to identify factors that influence these doses.

Ethiopia’s radiation protection and nuclear safety are regulated by Proclamation No. 1025, 2017 [18]. The framework includes some requirements for X‐ray equipment but does not address the need to optimize patient protection in medical exposures by implementing of DRLs [19]. To strengthen the legislation, it was considered necessary to measure incident air kerma (IAK) and offer guidance to national and local authorities and to clinicians on how to use DRLs as an effective tool for managing patient radiation doses in line with clinical goals.

The study aimed to determine the third quartile (75th percentile) of entrance surface air kerma (ESAK) for pediatric patients undergoing CXR examinations in 20 hospitals and clinics in Ethiopia and to use the obtained dose data for establishing the first‐ever national diagnostic reference levels (NDRLs) for the country.

## 2. Materials and Methods

### 2.1. Selection of Hospitals

To obtain a nationally representative sample, facilities were randomly selected across the country to capture radiographic imaging equipment from different manufacturers of X‐ray machines, taking into account regional differences in equipment availability and clinical practice. Facilities that did not routinely perform chest radiography on pediatric patients were excluded from the study. This selection procedure led to the inclusion of 20 radiography machines from 20 different facilities.

For hospitals equipped with more than one radiographic unit, one unit was specifically selected for assessment. The unit selected was the primary system routinely used for pediatric CXR examinations and was therefore considered to be the most representative of institutional practice and exposure conditions relevant to the objectives of the study. The inclusion of multiple units in the same hospital was avoided as pediatric imaging protocols are usually standardized at institutional level; including additional units would likely result in clustering effects without providing independent and clinically meaningful information.

### 2.2. DRL Surveys

ICRP publication 135 recommends weight bands for establishing pediatric DRL values for examinations of the trunk, while also stating that age bands grouped around ages of 0, 1, 5, 10, and 15 years can be used if age is the only available measure [20]. Thus, in the present study, pediatric age ranges of 5–< 10 years and 10–< 15 years were used to determine NDRLs in CXR examinations, reflecting local practice constraints and the absence of reliable patient weight data across all facilities. Although weight‐based grouping allows precise dosage estimation, age‐based categories are still extensively utilized in national DRL studies, especially in low‐ and middle‐income countries where systematic weight recording is not practicable. These classifications have been supported by current national studies and align with the European pediatrics DRL framework [21–23]. The ICRP stated that age bands grouped around ages of 0, 1, 5, 10, and 15 years can be used if age is the only available measure [20]. Thus, due to limitations in routine recording of pediatrics patients’ weight across the selected facilities, age‐based categories were used in the present study. However, using age instead of weight presents a significant methodological limitation. Children in the same age group may differ significantly in terms of chest thickness, body weight, and body habitus. Because of this, children of various sizes may fall into an age‐based category, which would increase dose variability within the group. When comparing current NDRLs to worldwide DRLs, the prospective influence of age‐based grouping must also be considered. Comparisons are most meaningful when the patient grouping method, dose quantity, projection, equipment type, and examination protocol are comparable. Accordingly, data collection focused on two pediatric age groups: 5–< 10 years and 10–< 15 years. Data were collected from 500 (5–10 years) and 475 (10–< 15 years) children, a total of 975 children; 56.3% were males. Nearly half of the CXR requests were for pneumonia (*n* = 494), followed by pulmonary tuberculosis (*n* = 176), cardiac illness (*n* = 111), trauma *n* = 77), hyperactive airway disease (HAAD) (*n* = 29), FB (*n* = 8), meconium aspiration syndrome (MAS) (*n* = 5), and PTB (*n* = 176) (see Table 1).

**TABLE 1:** Clinical characteristics of pediatric patients undergoing X‐ray examination.

Row labels	Count of clinical data
B asthma	29
Cardiac	111
FB	8
MAS	5
Medical checkup	17
Pleural effusion	19
Pneumonia	494
Perioperative workup	37
PTB	176
Scoliosis	1
Trauma	77
Tumor	1
Grand total	975

### 2.3. Sample Size and Sampling Technique

Data collection focused on two pediatric age groups: 5–< 10 years and 10–< 15 years. Unfortunately, sufficient data for children under 5 years of age have not been collected. In accordance with the recommendations of the ICRP [20] for patient dose surveys requiring at least 25 patients per hospital, a purposive sampling method was used. Only patients whose radiographic images had been reviewed and accepted by a radiologist were included in the study. This resulted in a total of 500 patients in the age group 5–< 10 years and 475 patients in the age group 10–< 15 years.

### 2.4. Data Collection Procedure

Data collection was conducted with a questionnaire prepared in English that was distributed to radiographers working in the selected hospitals and clinics. The questionnaires collected information about the X‐ray units, and completed forms were reviewed for accuracy and consistency before being retrieved from each facility.

Tube output (O/P) measurements were conducted in all participating hospitals, clinics, and diagnostic centers using a calibrated Unfors RaySafe Xi dosimeter under scatter‐free conditions. The measurements were performed at 80 kVp and 10 mAs, with a focus‐to‐detector distance (FDD) of 100 cm. The measured tube output was expressed as air kerma per unit mAs (mGy/mAs) and used to estimate the IAK at the patient entrance surface based on the clinical exposure parameters. During patient examinations, anthropometric and exposure parameters, including tube voltage (kVp), tube current–time product (mAs), and focus‐to‐skin distance (FSD), were recorded for each patient undergoing the specified diagnostic procedure.

IAK at the patient entrance surface was used as the primary dosimetric quantity and was converted to ESAK by applying an appropriate backscatter factor (BSF). The use of ESAK was based on its suitability for patient dose assessment in conventional radiography and its feasibility under the equipment and resource conditions of the participating facilities.

Although ICRP Publication 135 recommends recording both dose–area product (DAP) and ESAK, in the present study, only ESAK was used due to equipment and practical constraints [20].

The X‐ray units included in this study were not equipped with calibrated DAP meters, which limited the routine acquisition of reliable DAP measurements. In contrast, ESAK could be estimated using calibrated dosimetric measurements, tube output, and routinely recorded exposure parameters. ESAK is also a well‐established dosimetric quantity for patient dose assessment in conventional radiography and has been widely reported in DRL studies, facilitating comparison with published national and international DRLs. Therefore, ESAK was considered an appropriate and feasible dosimetric quantity for the objectives of this study.

The ESAK was calculated using the following equations:

(1) ESAK=OPmAskVp802100FSD2BSF,

where (O/P) is the measured tube output at 100 cm (mGy/mAs), mAs is the clinical tube current–time product, and FSD is the focus‐to‐skin distance in cm. BSF is the BSF corresponding to the relevant beam quality and irradiation conditions. A BSF of 1.35 was used in this study to convert IAK to ESAK.

The BSF of 1.35 was used in accordance with internationally accepted practice for general diagnostic radiography, indicating that the selected value was based on published reference studies and that BSF values for general diagnostic radiography typically fall within the range of 1.3–1.4, depending on beam quality, field size, and radiation geometry [20, 24, 25]. Because patient‐specific BSF measurements were not feasible under the conditions of this multicenter study, a value of 1.35 was selected as a representative value within this range. The same BSF was applied consistently across the participating facilities to provide a standardized approach to ESAK estimation and to facilitate comparison of patient dose distributions and DRLs across facilities and with previously published studies.

### 2.5. Data Processing and Analysis

The collected data were assessed for reliability, then entered, cleaned, and analyzed using SPSS Version 27. Statistical summaries were adjusted to determine the mean, maximum, and minimum values of the recorded exposure parameters. In addition, the mean, maximum, minimum, and third quartile values of the calculated ESAK were presented in tables to enable comparison with national and international reference values. For each diagnostic examination, ESAK values were compiled into procedure‐specific datasets. The data were first checked for completeness and plausibility, and descriptive statistics were generated. In accordance with ICRP recommendations, the median values were calculated from representative sample of patients within an agreed age group for each facility [20]. The 75th percentile of the medians of the dose distributions across facilities was used to calculate the NDRLs. The NDRL value was compared to recently published DRLs from other countries.

### 2.6. Ethical Considerations

The study respected ethical principles to ensure the rights and dignity of the participants. Clear and detailed explanations of the purpose of the study were provided to the carers. Confidentiality was maintained by excluding any personal identifiers such as names from the data.

## 3. Results and Discussion

We analyzed anthropometric data from 975 pediatric patients who had chest radiographic examinations across 20 different hospitals and clinics in Ethiopia. Tables 2 and 3 present center‐level mean, minimum, and maximum values for kVp, mAs, height, weight, body mass index (BMI), grid use, and FSD, together with the mean and median ESAK. The minimum and maximum values represent the lowest and highest center‐specific mean values, respectively, rather than individual patient measurements. Therefore, they do not represent the minimum or maximum exposure received by an individual child. The findings demonstrate substantial intercenter variation in patient anthropometric characteristics and radiographic exposure parameters, particularly among children aged 10–< 15 years.

**TABLE 2:** The mean, range (min, max) patient anthropometric characteristics, exposure parameters, and ESAK (mean, median) values for pediatrics 5–< 10 years of age.

Centers	O/P	kVp mean (min–max)	mAs (mean, min–max)	Height (cm) mean (min–max)	Weight (kg) mean (min–max)	BMI (kg/m^2^) mean (min–max)	Grid (yes/no)	FSD mean (min–max)	ESAK (mean, median)
SU	0.051	110 (80–130)	0.8 (0.5–1.0)	110 (94–130)	15 (8–25)	15 (12–22)	Yes	150 (110–150)	0.051, 0.05
PO	0.043	110 (100–130)	0.9 (0.49–1.0)	120 (100–130)	15 (9–16)	14 (12–17)	Yes	100 (100–100)	0.043, 0.04
HG	0.061	120 (80–148)	0.8 (0.6–1.0)	130 (113–148)	28 (15–44)	18 (12–28)	Yes	120 (100–120)	0.061, 0.06
AH	0.047	100 (70–140)	0.7 (0.6–1.0)	125 (113–145)	25 (13–45)	17 (10–25)	Yes	120 (100–150)	0.047, 0.05
BH	0.031	120 (80–135)	0.9 (0.8–1.0)	130 (107–141)	28 (18–39)	17 (12–22)	Yes	100 (100–100)	0.031, 0.03
BLH	0.048	80 (60–150)	0.8 (0.6–1.0)	125 (100–155)	25 (15–38)	18 (12–24)	Yes	150 (100–150)	0.048, 0.05
ICMC	0.067	100 (60–128)	0.8 (0.6–1.0)	120 (100–128)	20 (15–27)	15 (12–18)	Yes	100 (100–100)	0.067, 0.07
ADH	0.073	100 (90–132)	0.7 (0.5–1.0)	120 (90–132)	25 (18–35)	22 (15–33)	Yes	120 (100–120)	0.073, 0.07
FH	0.051	80 (50–200)	0.8 (0.5–1.0)	115 (100–140)	28 (18–50)	20 (12–30)	Yes	150 (100–150)	0.051, 0.05
DrE	0.012	100 (60–135)	0.8 (0.6–1.0)	125 (110–135)	25 (16–32)	18 (12–25)	Yes	100 (100–100)	0.012, 0.01
KH	0.051	100 (60–128)	0.8 (0.6–1.0)	120 (100–128)	22 (15–27)	16 (12–22)	Yes	100 (100–100)	0.051, 0.05
LH	0.087	87 (82–94)	0.8 (0.7–0.9)	92 (79–95)	20 (14–24)	23 (19–28)	No	150 (145–150)	0.087, 0.09
MSH	0.034	115 (100–160)	1 (0.9–1.0)	120 (100–160)	30 (20–35)	20 (15–25)	Yes	150 (90–150)	0.034, 0.03
KGH	0.027	100 (100–133)	0.9 (0.7–1.0)	120 (107–155)	22 (17–27)	15 (12–18)	Yes	150 (150–150)	0.051, 0.05
SPH	0.051	100 (100–133)	0.6 (0.5–0.7)	120 (108–133)	22 (17–27)	15 (12–18)	Yes	120 (100–150)	0.051, 0.05
TGH	0.045	80 (50–145)	0.6 (0.5–0.7)	120 (95–145)	30 (18–44)	20 (12–30)	Yes/No	120 (100–150)	0.045, 0.04

**TABLE 3:** The mean, range (min, max) patient anthropometric characteristics, exposure parameters, and ESAK (mean, median) values for pediatrics between 10 and < 15 years of age.

Centers	O/p	KV mean (min–max)	mAs mean (min–max)	Height (cm) mean (min–max)	Weight (kg) mean (min–max)	BMI (kg/m^2^) mean (min–max)	Grid yes/no	FSD mean (min–max)	ESAK (mean, median)
SC	0.051	77.6 (64–94)	1.63 (0.5–11.6)	134.08 (115–155)	33.52 (20–48)	18.7 (11.89–31.25)	Yes	127.7 (106–139)	0.07,0.03
PO	0.043	65 (65–65)	2 (2–2)	118.36 (110–125)	23.36 (18–30)	16.80 (11.52–22.68)	Yes	141.2 (100–180)	0.06, 0.04
HG	0.061	65.96 (63–69)	4.54 (3.15–5.52)	144.8 (125–162)	42.4 (28–60)	20.32 (13.28–31.82)	Yes	158.4 (120–180)	0.16, 0.13
AH	0.047	93.36 (40–150)	4.05 (0.8–16)	144.72 (130–170)	25.68 (18–35)	16.91 (11.76–21.42)	Yes	124.9 (79–153)	0.20, 0.15
BH	0.031	55.96 (53–60)	9 (9–9)	144.12 (131–156)	43.68 (33–54)	20.93 (15–23.30)	Yes	130	0.15,0.14
BLH	0.048	60.88 (56–94)	12.36 (2–16)	142.28 (129–170)	39.84 (25–68)	19.68 (15–23.8)	Yes	150	0.30,0.29
ICMC	0.067	77.6 (64–94)	1.63 (0.5–11.6)	134.08 (115–155)	33.52 (20–48)	18.71 (11.89–31.25)	Yes	140.8 (120–150)	0.09,0.04
ADH	0.073	55.24 (50–60)	17.46 (12–22.5)	135.76 (127–146)	40.16 (32–59)	21.89 (16.59–28.06)	Yes	70.48 (64–76)	1.68,1.72
FH	0.051	75.2 (50–100)	3.89 (0.48–6.4)	133.72 (102–142)	37.30 (0.4–50)	20.41 (15.75–32.29)	Yes	132.62 (86–138)	0.07, 0.09
DrE	0.012	64.2 (60–65)	2.98 (2‐5‐3)	136.60 (125–152)	35.04 (20–55)	18.80 (10.97–33.57)	Yes	88.48 (86–90)	0.04, 0.04
KH	0.051	62.24 (60–70)	13.12 (12–16)	126.33 (122–151)	36.96 (23–70)	21.28 (15.97–25.82)	Yes	85.66 (82–89)	0.75, 0.64
LH	0.087	86.88 (66–89)	2.44 (2–2.5)	96.12 (90–102)	25.84 (22–30)	27.92 (25.44–27.92)	Yes	86.88 (66–89)	0.20,0.20
MSH	0.034	115 (115–115)	10 (10–10)	128.8 (100–170)	38.92 (17–60)	23.31 (16.53–33.05)	Yes	135.12 (130–140)	0.53, 0.53
KGH	0.027	104 (104–104)	12.6 (9–99)	149.52 (140–165)	40.04 (5–52)	17.91 (2.16–22.82)	Yes	87.28 (82–93)	1.06,0.73
SPH	0.051	55.32 (53–60)	4.36 (2–9)	113.64 (108–133)	33.88 (22–52)	26.43 (15.26–35.15)	Yes	125.8 (91–150)	0.09,0.10
TGH	0.045	63.33 (50–70)	6.84 (0.48–10)	140.76 (120–165)	49.76 (33–70)	25.03 (18.10–31.63)	Yes	150	0.15,0.13
VC	0.024	62.24 (60–70)	13.12 (12–16)	131 (120–151)	36.8 (23–50)	21.24 (15‐97‐27.10)	Yes	85.66 (82–89)	0.36, 0.30
Y12H	0.025	58.04 (56–60)	6.93 (6.4–8.2)	143.6 (130–160)	40.2 (30–55)	19.33 (14.26–23.11)	Yes	135.68 (131–139)	0.07, 0.07
YCH	0.027	49 (49–49)	9 (9–9)	135.88 (125–183)	30.64 (26–39)	16.78 (8.65–20.18)	Yes	150	0.66, 0.66

For children aged 5–< 10 years, the center‐specific mean FSD ranged from 77 cm to 150 cm. Mean kVp ranged from 50 to 100 kVp, while mean mAs ranged from 0.160 to 1.00 mAs. Mean BMI ranged from 11 to 23 kg/m^2^. Most centers reported grid use, except the two WH and LH. The center‐level mean ESAK ranged from 0.012 to 0.087 mGy. These findings indicate considerable variation in radiographic practice among centers despite the relatively narrow age range.

The NDRLs from this study for age groups and X‐ray projections are presented in Table 3. Accordingly, the FSD used for CXR among the pediatric 10–< 15 years of age ranged from a minimum of 64 cm to a maximum of 180 cm. All of the facilities used a grid. The minimum and maximum values of kV and mAs for similar age groups are 10 and 150 keV, and 0.48–90 mAs, respectively. The minimum and maximum mean values of the BMI for all centers are 16.78 and 26.43 kg/m^2^, respectively.

The selection of optimal tube potential and tube loading in chest radiography has been extensively debated in the literature [26, 27]. Due to the excellent subject contrast of this anatomical structure and the need to minimize motion artifact, it is recommended to use low mAs and high kVp settings for pediatric chest radiography. Specifically, kVp values of 60–65 are advised for neonates, 70–100 for children aged 1–5 years, and 100–120 for ages 5–15 years [26]. Studies strongly discourage the use of tube voltages below 60 kVp for pediatric patients [19, 27, 28]. For mAs, the recommended range is 1–2 mAs for children aged 0–5 years and 2–4 mAs for those aged 5–15 years [19].

However, in this study, substantial variation in kVp and mAs was observed across facilities and age groups. For children aged 5–< 10 years, the mean kVp values across facilities ranged from 80 to 120 kVp, while the recorded individual exposure ranges extended from 50 to 200 kVp. The corresponding mean mAs values ranged from approximately 0.6–1.0 mAs, with recorded values ranging from 0.49 to 1.0 mAs. For children aged 10–< 15 years, the mean kVp values ranged from 49 to 115 kVp, with recorded exposure values ranging from 49 to 150 kVp, while mean mAs values ranged from 1.63 to 17.46 mAs. These findings demonstrate considerable variation in exposure selection between facilities and indicate that some facilities used tube potentials below the recommended 100–120 kVp range for children aged 5–15 years, while mAs values in several facilities were also higher than the recommended 2–4 mAs range. This variation suggests a lack of standardized exposure protocols and emphasizes the need for optimized pediatric chest radiography techniques. The variation in exposure parameters was also reflected in the ESAK values. Among children aged 5–< 10 years, facility‐specific mean ESAK values ranged from 0.012 to 0.087 mGy, with the highest mean ESAK of 0.087 mGy observed at LH, where the mean kVp, mAs, and FSD were 87 kVp, approximately 0.8 mAs, and 150 cm, respectively. In the 10–< 15‐year age group, mean ESAK values ranged from 0.04 to 1.68 mGy, with the highest value of 1.68 mGy recorded at ADH, corresponding to a mean kVp of 55.24 kVp, mAs of 17.46, and FSD of 70.48 cm. The markedly higher ESAK at ADH is consistent with the combination of relatively low kVp, high mAs, and short FSD, all of which can contribute to increased patient entrance surface dose. These findings highlight important deviations from optimized exposure techniques and support the need for standardized pediatric radiographic protocols.

The selection of kVp and mAs for CXRs must be scientifically optimized based on the patient’s BMI to achieve the dual goals of maintaining diagnostic image quality and minimizing radiation exposure [29]. This approach ensures sufficient X‐ray penetration for higher BMI patients while avoiding overexposure in lower BMI individuals. In the present study, considerable variation in BMI was observed across facilities. For children aged 5–< 10 years, the mean BMI values ranged from 14 to 23 kg/m^2^, while for children aged 10–< 15 years, they ranged from 16.78 to 27.92 kg/m^2^. The observed variation in ESAK in relation to patient BMI and exposure parameters suggests inconsistent optimization of exposure techniques according to patient size. Therefore, establishing BMI‐ or size‐specific exposure protocols and technique charts could improve standardization of pediatric chest radiography and support radiation dose optimization.

Our survey found that the third quartile of radiation dose used in Ethiopia for pediatric chest radiographs is 0.07 mGy for 5–< 10 years and 0.53 mGy for 10–< 15 years. The NDRL of 0.07 mGy for the 5–< 10‐year age group is below the previously reported Ethiopian LDRL of 0.17 mGy, whereas the NDRL of 0.53 mGy for the 10–< 15‐year age group is substantially higher than the reported LDRL of 0.17 mGy [19] and the European Union (0.1 mGy) [30]. The difference between the two age groups indicates that dose optimization remains particularly important for older pediatric patients, where substantial variation in mAs and other exposure parameters was observed.

We compared our results with published DRL data in pediatrics [19, 26, 27, 29–31]. We compared the proposed NDRLs with published pediatric DRL data [22, 30–35]. As shown in Table 4, the NDRL of 0.07 mGy for children aged 5–< 10 years is lower than the previously reported Ethiopian LDRL (0.17 mGy) and comparable international values, whereas the NDRL of 0.53 mGy for children aged 10–< 15 years is higher than the corresponding values reported in most comparable studies. The relatively high NDRL in the 10–< 15‐year age group may be partly explained by the wide variation in mAs, kVp, and FSD observed between facilities, including the use of relatively high mAs and short FSD at some centers. In particular, the 10–< 15‐year group showed mAs values as high as 17.46 mAs and FSD values as low as 64 cm, demonstrating substantial departures from consistent exposure practices. These findings indicate the need for greater standardization of pediatric chest radiography techniques. Furthermore, staff training and awareness of the technical aspects involved in CXR imaging play an important role in affecting patient radiation dose [32]. Scanning protocols vary across countries, largely depending on the operators′ training and experience levels. Abdulkadir et al. [33] highlighted that radiographers often lack sufficient knowledge regarding DRLs, particularly in applying local dose assessments and optimizing DRLs effectively.

**TABLE 4:** Comparison of the proposed NDRL of the current study to others’ DRLs.

Country	National and international DRL: ESAK (mGy)	
	Age group	
	5–< 10 years	10–< 15 years
Ethiopia Current study	0.07	0.53
Ethiopia (LDRL (2022)) [19]	0.17	0.17
Portugal [26]	0.08	0.1
	0.3	0.3
UNSCHR (2000) [33]	0.04	0.05
Austria [7]	0.06	0.08
European [30]	0.23	0.45
European [29]	0.1	0.1
Kenya [27]	0.07	0.09
Japan [32]	0.2	0.2

Strengthening radiologic technologist training, implementing standardized pediatric exposure charts, and regularly reviewing facility‐specific dose distributions against the national DRLs may therefore contribute to more consistent and optimized pediatric chest radiography practices in Ethiopia.

This study showed that the radiographic exposure settings employed varied considerably across the participating facilities, particularly in kVp, mAs, FSD, and patient size characteristics. Radiographers and medical radiologic technologists typically choose exposure parameters on their own, which may account for this disparity. This is consistent with a recent report that found significant differences in patient doses and exposure parameters both within and between hospitals [34]. In the present study, this variability was particularly evident among children aged 10–< 15 years, for whom mean mAs values ranged from 1.63 to 17.46 mAs and mean FSD values ranged from 64 to 150 cm. The 5–< 10‐year group also demonstrated variation in mean kVp (80–120 kVp) and mean FSD (100–150 cm). Such practices may contribute to variations in patient radiation dose. National DRLs can be used to address these disparities in exposure parameters. National DRLs provide benchmarks for identifying unusually high doses and encourage standardization and optimization of radiographic practices across radiology departments. A recent study on pediatric DRLs proposes weight‐stratified local DRLs, as recommended by the ICRP, with KAP values ranging from 4 mGy cm^2^ to 884 mGy cm^2^ for different examinations [35]. Establishing DRLs for radiographs from a weight‐based patient study provides reference values for image optimizations, according to a new study on DRL establishment for pediatric radiographic examinations [35]. The reported KAP‐based DRLs reflect optimized pediatric chest radiography practices, including appropriate collimation and age‐ or weight‐adapted exposure parameters [35]. However, direct quantitative comparison with the present study is not appropriate because the current study used ESAK rather than KAP as the dose metric. Although a direct quantitative comparison is therefore not possible, the present study provides age‐specific ESAK‐based national reference values that can be used to assess dose variation and support optimization of pediatric chest radiography in Ethiopia.

This represents a methodological limitation of the present study, as the absence of field size or KAP measurements restricts direct comparison with international DRLs [36]. Future studies should incorporate systematic recording of beam collimation and KAP to enable more robust DRL comparisons and image quality.

### 3.1. Strengths and Limitations of the Study

This study presents a comprehensive assessment of pediatric chest radiographic practices across 20 hospitals and clinics in Ethiopia, analyzing data from 975 pediatric patients. A major strength of the study is its large, multicenter sample size, which enhances the generalizability of the findings within the national context. The inclusion of detailed technical parameters such as kVp, mAs, FSD, BMI, and grid usage enables a robust evaluation of exposure settings. Moreover, the comparison with both local and DRLs offers critical insight into the current status of radiological practice in Ethiopia, pinpointing areas that deviate from best practice and advocating for improved standardization through national DRLs and staff training.

However, the study also faces certain limitations. The main limitation of this survey is the use of age as a grouping parameter, instead of weight as recommended by ICRP [2, 37]. The minimum and maximum values presented are averages at the facility level rather than individual patient doses, which limits patient‐specific interpretation. The retrospective nature of the data introduces potential inconsistencies in how information was recorded across sites. Notably, the study does not evaluate image quality, making it difficult to determine whether higher doses resulted in better diagnostic outcomes. Additionally, variability in radiographic technique, driven by operator discretion, reveals a lack of standardized exposure protocols. The absence of more comprehensive dose metrics, such as DAP, further constrains the analysis. Lastly, while the study identifies training gaps, it does not directly assess radiographers’ knowledge or practices, which would strengthen recommendations for educational interventions.

## 4. Conclusion

This study, conducted in 20 Ethiopian hospitals and clinics and involving 975 pediatric patients, provides valuable insight into radiation dose levels and radiographic practices in pediatric chest radiography. The findings show substantial variation in ESAK and exposure parameters across facilities, particularly in kVp, mAs, and FSD. The proposed NDRLs were 0.07 mGy for children aged 5–< 10 years and 0.53 mGy for those aged 10–< 15 years, demonstrating different dose distributions between the two age groups and highlighting the need for continued optimization, particularly among older pediatric patients.

Establishing national DRLs is a critical step toward harmonizing pediatric chest radiography practices across the country. The proposed NDRLs provide national benchmarks against which facility‐specific doses can be compared and unusually high doses identified. Their implementation should be accompanied by standardized pediatric exposure protocols, appropriate technique charts, regular dose monitoring, and continued training of radiographers and medical radiologic technologists.

Future updates to pediatric CXR DRLs should incorporate larger multicenter datasets, weight‐ or size‐based categorization, KAP/DAP and field‐size measurements, and image‐quality assessment to strengthen dose optimization and facilitate comparison with international DRLs. Sustained participation of healthcare institutions in dose‐monitoring programs and periodic review of the NDRLs will be essential for maintaining and improving radiation protection practices in pediatric radiography.

## Funding

The authors disclosed receipt of the following financial support for the research and authorship of this article: This work was supported by Addis Ababa University, College of Health Science. This research was financially supported by Addis Ababa University, the Office of the President for Research and Technology Transfer (VPRRT).

## Ethics Statement

With protocol no. 108/22/Radio, the Institutional Research Ethics Review Board of Addis Ababa University’s College of Health Sciences (AAU‐CHS‐IRB) provided ethical approval. Because there was no direct patient contact during data collection, the Department of Radiology Research and Ethics Committee also accepted the waiver of informed consent. Additionally, the study was conducted in compliance with the Helsinki Declaration’s pertinent rules and regulations.

## Conflicts of Interest

The authors declare no conflicts of interest.

## Data Availability

The data that support the findings of this study are available from the corresponding author upon reasonable request.
